# A Pilot Study of Saliva MicroRNA Signatures in Children with Moderate-to-Severe Traumatic Brain Injury

**DOI:** 10.3390/jcm13175065

**Published:** 2024-08-27

**Authors:** Robert Ciancaglini, Ann S. Botash, Veronica Armijo-Garcia, Kent P. Hymel, Neal J. Thomas, Steven D. Hicks

**Affiliations:** 1 Department of Pediatrics, Penn State Health Children’s Hospital, Hershey, PA 17033, USA; rciancaglini@pennstatehealth.psu.edu (R.C.); nthomas@pennstatehealth.psu.edu (N.J.T.); 2 Department of Pediatrics, SUNY Upstate Golisano Children’s Hospital, Syracuse, NY 13210, USA; botasha@upstate.edu; 3 Department of Pediatrics, UT Health San Antonio, San Antonio, TX 78229, USA; mirelesv@uthscsa.edu

**Keywords:** traumatic brain injury, pediatrics, TBI, trauma

## Abstract

**Background/Objectives**: Traumatic brain injury (TBI) is a leading cause of death and disability in children. Currently, no biological test can predict outcomes in pediatric TBI, complicating medical management. This study sought to identify brain-related micro-ribosomal nucleic acids (miRNAs) in saliva associated with moderate-to-severe TBI in children, offering a potential non-invasive, prognostic tool. **Methods**: A case-control design was used, enrolling participants ≤ 18 years old from three pediatric trauma centers. Participants were divided into moderate-to-severe TBI and non-TBI trauma control groups. Saliva samples were collected within 24 h of injury, with additional samples at 24–48 h and >48 h post-injury from the TBI group. miRNA profiles were visualized with partial least squares discriminant analysis (PLSDA) and hierarchical clustering. Mann–Whitney testing was used to compare miRNAs between groups, and mixed models were used to assess longitudinal expression patterns. DIANA miRPath v3.0 was used to interrogate the physiological functions of miRNAs. **Results**: Twenty-three participants were enrolled (14 TBI, nine controls). TBI and control groups displayed complete separation of miRNA profiles on PLSDA. Three miRNAs were elevated (adj. *p* < 0.05) in TBI (miR-1255b-5p, miR-3142, and miR-4320), and two were lower (miR-326 and miR-4646-5p). Three miRNAs (miR-3907, miR-4254, and miR-1273g-5p) showed temporal changes post-injury. Brain-related targets of these miRNAs included the glutamatergic synapse and *GRIN2B*. **Conclusions**: This study shows that saliva miRNA profiles in children with moderate-to-severe TBI may differ from those with non-TBI trauma and exhibit temporal changes post-injury. These miRNAs could serve as non-invasive biomarkers for prognosticating pediatric TBI outcomes. Further studies are needed to confirm these findings.

## 1. Introduction

Traumatic brain injury (TBI) is the leading cause of death and long-term disability for children in the United States [1]. Over 6000 children die each year from TBI [2], and more than 61% of children who sustain a moderate-to-severe TBI will suffer from long-term disabilities [3], including substantial cognitive, social, behavioral, and physical impairments. There is currently no biologic test that predicts outcomes in pediatric TBI. Such a test could guide medical management decisions for pediatricians and provide valuable information for worried parents.

Epi-transcriptional biomarkers, such as micro-ribosomal nucleic acids (miRNAs), may provide a novel window into the injured brain [4]. Extracellular transport of miRNAs (through exosomes and micro-vesicles) is an established mechanism by which cells communicate, altering gene expression in surrounding tissues [5]. This unique feature allows one to sample nucleic acid material from cranial nerves through the non-invasive collection of saliva [6]. Previous studies of saliva miRNA in children with mild TBI showed that two-thirds of saliva miRNAs are brain-related [7].

Many blood-based TBI biomarkers [8,9] can be measured through non-invasive saliva collection and may be used to aid diagnosis of mild TBI [10,11,12]. The physiologic specificity of miRNA signaling also translates into prognostic potential [13], which may have advantages when compared with protein biomarkers [10,12]. For example, glial fibrillary acidic protein (GFAP), a commonly studied TBI biomarker [14,15], generally requires severe neurovascular injury and apoptotic cell death to cross the blood–brain barrier [16]. In comparison, miRNAs can be released by live neurons and glia that are actively responding to oxidative stress, axonal shearing, or cerebral edema. GFAP levels were recently approved by the Food and Drug Administration to determine the necessity of brain imaging in adults with TBI [17], but have age-specific variations that may impair clinical application in children [18], and have not been shown to predict medical outcomes (i.e., morbidity or mortality).

The goal of this study was to determine if brain-related miRNAs in the saliva of children are associated with moderate-to-severe TBI and examine whether salivary miRNAs display longitudinal changes in expression during the acute recovery phase. We hypothesized that saliva miRNAs could provide sufficient biologic granularity to discern this critical prognostic information.

## 2. Materials and Methods

This study received ethical approval from the institutional review board at the Penn State College of Medicine and SUNY Upstate Medical University. Written, informed consent was obtained from the caregivers of all participants.

The study employed a case-control design. Participants ≤ 18 years of age were enrolled at three level one pediatric trauma centers between September 2019 and June 2021. Participants were assigned to one of two groups: the moderate-to-severe TBI group (sTBI) or the control group. The TBI group consisted of 14 children admitted to the hospital for a moderate-to-severe TBI as defined by the Mayo classification system [19]. The control group consisted of nine children admitted to the hospital with non-TBI trauma or polytrauma (i.e., with no traumatic injuries above the level of the neck). Exclusion criteria for both groups included history of previous TBI, active periodontal disease, oropharyngeal trauma, epilepsy, intellectual disability, concurrent upper respiratory infection, congenital brain malformation, or history of hypoxic-ischemic encephalopathy (HIE). A post-hoc power analysis determined that this sample size provided 82% power to detect a 1.75-fold difference among 25 miRNAs (α = 0.04), based on a mean standard deviation in miRNA read counts of 0.7.

After obtaining informed consent, medical record review was used to obtain details from the physical exam at the time of admission (e.g., Glasgow Coma Score for assessment of consciousness), as well as age, sex, ethnicity, psychiatric history, medical history, and medications (both home medications and those given during hospital admission). Details of traumatic injury were collected, including time since injury, mechanism of injury, location of injury, and associated symptoms (e.g., amnesia, emesis, seizures, weakness, loss of consciousness). If clinically indicated, results of cranial imaging (i.e., computed tomography or magnetic resonance imaging) were also extracted from the electronic medical record. REDCap was used to record de-identified participant data across all three centers.

Sub-lingual saliva was collected from all participants using a swab technique within 24 h of injury. For the sTBI group we collected additional samples 24–48 h after injury (*n* = 9) and >48 h after injury (*n* = 5). Saliva was collected after tap water rinse (or oral hygiene regimen provided by nurse for intubated patients) using P-157 nucleic acid stabilization swabs (DNA Genotek, Ottawa, ON, Canada). Samples were stored at −20 °C and processed by the Penn State Genomic Sciences Core Facility. Saliva RNA was purified as we have previously reported [20]. RNA quality was assessed using the Agilent 2100 Bioanalyzer (Agilent Technologies, Inc., Santa Clara, CA, USA). The 260/280 ratio and the 260/230 ratio were used to assess RNA quality, and RNA extraction was repeated for any samples where the ratios were less than 2.0. RNA was quantified on an Illumina HiSeq instrument at ~10 million reads per sample. Mature and immature miRNA counts were determined through alignment to the human genome in Partek Flow with the Bowtie1 algorithm. Reads were examined for sequence bias, position bias, and guanosine/cytosine bias in Partek Flow. Raw reads from each sample were quantile normalized to control for inter-individual variation in saliva collection volumes and total RNA content. Sparse miRNAs were filtered, and the features present in raw counts >10 in >50% of samples were interrogated.

Medical and demographic traits were compared between groups with a Student’s *t*-test or chi-square test as appropriate. Differences in the total saliva miRNA profile between groups were visualized with a two-dimensional partial least squares discriminant analysis (PLSDA). Based on a power analysis, 25 miRNAs with the highest variable importance in projection (VIP) on PLSDA were used for downstream analyses. First, hierarchical clustering of individual samples (Ward clustering algorithm, Euclidean distance metric) was performed. Next, Mann–Whitney U-testing was used to compare salivary levels of the 25 miRNAs between control samples and initial samples (<24 h post-injury) obtained from participants with sTBI. Benjamini Hochberg multiple testing correction was applied. Next, total miRNA profiles were compared across three timepoints (0–24 h post-injury, 24–48 h post-injury, and >48 h post-injury) for participants with sTBI using PLSDA. miRNA candidates were identified using VIP scores, and mixed models were used to examine the effect of collection time on levels of miRNA while controlling for participant ID as a clustering variable. Physiologic functions of miRNAs that differed between sTBI and control samples and miRNAs that varied with time of collection after sTBI were determined using DIANA miRPath v3.0 [21]. Putative transcript targets for each miRNA candidate were determined with the microT-CDS algorithm (threshold > 0.90, *p* < 0.02), and Fisher’s exact testing with false detection rate correction was used to identify Kyoto Encyclopedia of Genes and Genomes (KEGG) pathways targeted by the miRNAs with greater frequency than that expected by chance alone. Intersecting gene targets and pathway targets were also reported.

## 3. Results

### 3.1. sTBI vs. Control Group

A total of 23 patients were enrolled across all three sites, including 14 sTBI patients and nine controls (Table 1). The average age of participants in the control group (122 months ± 64) was slightly older (*p* = 0.077) than those in the sTBI group (67 months ± 52). The most common mechanism of injury among sTBI participants was falling (7/14; 50%; Table 2). Half of sTBI participants sustained a subdural hematoma (SDH). Bony injury was the most common injury for participants in the control group (4/9; 44%; Table 3). Two participants in the sTBI group were intubated at the time of saliva collection. There was no difference between groups in sex or weight.

**Figure 1:**
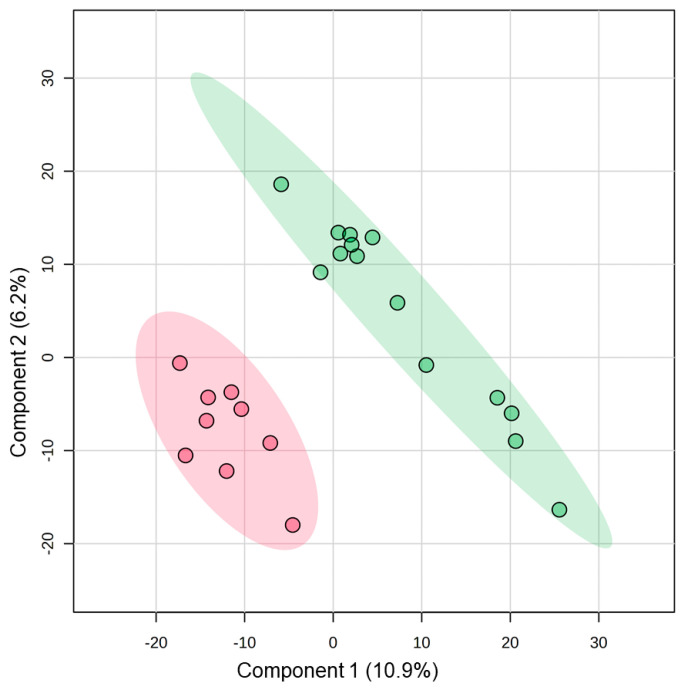
The two-dimensional partial least squares discriminant analysis (PLSDA) plots individual samples from participants with sTBI (green; *n* = 14) and controls (red; *n* = 9) based on total salivary miRNA profile. The PLSDA revealed complete separation of groups while accounting for 17.1% of the variance in the data. Shaded ovals represent 95% confidence interval.

**Figure 2:**
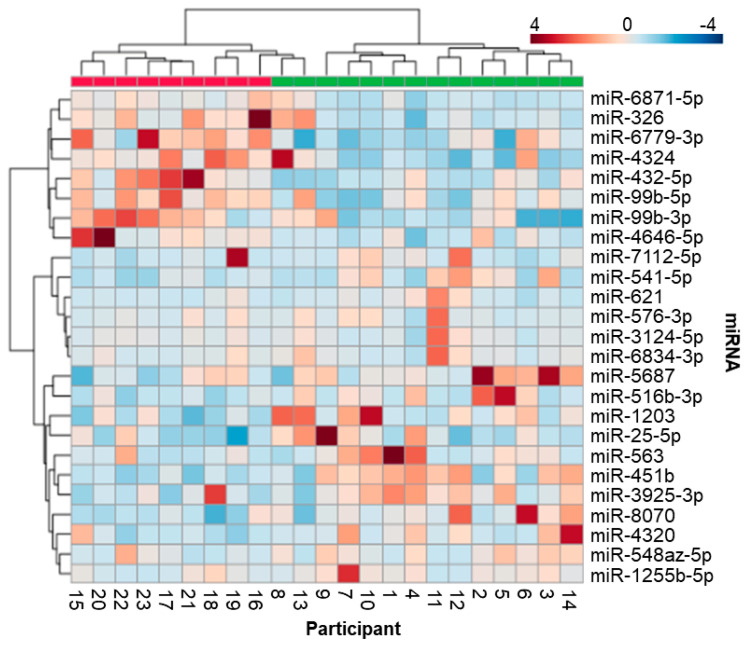
The heatmap shows salivary levels of 25 candidate miRNAs across 14 participants with sTBI (green) and nine controls. Hierarchical clustering of participants and miRNAs was achieved with the Ward clustering method and a Euclidean distance measure. Blue boxes represent relative down-regulation, and red boxes represent up-regulation.

**Figure 3:**
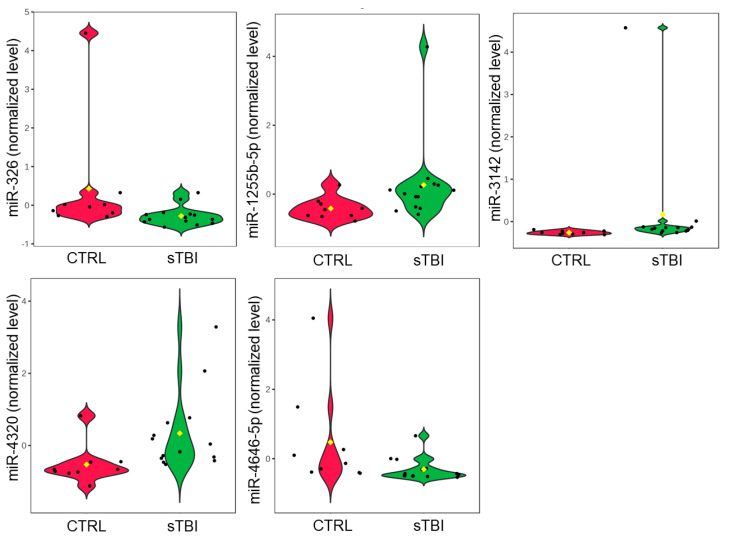
The violin plots display quantile normalized levels for the five miRNA candidates that displayed significant differences (adjusted *p* < 0.05) between 14 participants with sTBI (green) and nine controls (red) on Mann–Whitney U-testing. Salivary levels of miR-326 (U = 102.5, *p* = 0.013, adj. *p* = 0.041) and miR-4646-5p (U = 102.0, *p* = 0.015, adj. *p* = 0.030) were lower among partricipants with sTBI, whereas levels of miR-1255b-5p (U = 24.0, *p* = 0.015, adj. *p* = 0.015), miR-3142 (U = 13.0, *p* = 0.00087, adj. *p* = 0.021), and miR-4320 (U = 4320, *p* = 0.0020, adj. *p* = 0.049) were higher in the saliva of participants with sTBI. Mean values (yellow diamond) are shown. Individual black dots represent individual participant miRNA levels.

Raw mature miRNA counts did not differ (*p* > 0.05) between control samples (*n* = 9, 645,757, IQR: 183,735), initial sTBI samples (*n* = 14, 471,044, IQR: 221,404), or follow-up sTBI samples (*n* = 14, 331,191, IQR: 192,257; Table S1). There were 2133 miRNAs with raw counts ≥ 10 in at least half the samples, and 200 miRNAs were present in all samples. The most abundant miRNA was miR-203a-3p (4,189,612 raw reads across all samples). Total salivary miRNA profiles displayed prominent differences between sTBI and control samples. Visualization with two-dimensional PLSDA revealed complete separation of groups while accounting for 17.1% of the variance in the data (Figure 1). The 25 miRNAs with the highest VIP scores on PLSDA were examined across individual participants using hierarchical clustering. Hierarchical clustering of the 14 sTBI and nine control samples completely segregated the sTBI and control samples while demonstrating similarities in miRNA expression across individual participants (Figure 2). For example, participants 2, 5, 11, and 12 were closely clustered, and all suffered subdural hematomas. The hierarchical clustering plot also displays relationships between the 25 miRNAs with high VIP scores. There were eight miRNAs with relatively higher expression in the control group (clustered at the top) and seventeen miRNAs with relatively higher expression in the sTBI group (clustered at the bottom). Comparison of these 25 miRNAs across sTBI and control groups with Mann–Whitney U-testing showed that all 25 displayed nominal differences (raw *p* < 0.05) between initial sTBI samples and control samples, but only five withstood multiple testing corrections (adj. *p* < 0.05). Salivary levels of miR-326 (U = 102.5, *p* = 0.013, adj. *p* = 0.041) and miR-4646-5p (U = 102.0, *p* = 0.015, adj. *p* = 0.030) were lower among partricipants with sTBI, whereas levels of miR-1255b-5p (U = 24.0, *p* = 0.015, adj. *p* = 0.015), miR-3142 (U = 13.0, *p* = 0.00087, adj. *p* = 0.021), and miR-4320 (U = 4320, *p* = 0.0020, adj. *p* = 0.049) were higher in the saliva of participants with sTBI (Figure 3).

### 3.2. sTBI Patients over Time

**Figure 4:**
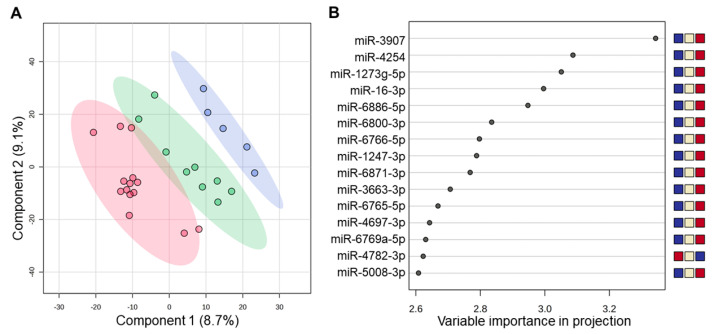
The two-dimensional partial least squares discriminant analysis (PLSDA) plots individual samples from participants with sTBI within 24 h of injury (red; *n* = 14), 24–48 h after injury (green; *n* = 9), and >48 h after injury (blue; *n* = 5) (**A**). The PLSDA achieved nearly complete separation of groups while accounting for 17.8% of the variance in the data. Shaded ovals represent 95% confidence interval. The variable importance in projection plot (**B**) displays the 15 miRNAs that contributed most to group separation on PLSDA. Blue circles denote VIP scores for each miRNA. The relative expression levels of each miRNA across the three time points are displayed in the colored squares, where blue denotes low expression and red denotes high expression. Note that most miRNAs increased in expression across the three time points.

**Figure 5:**
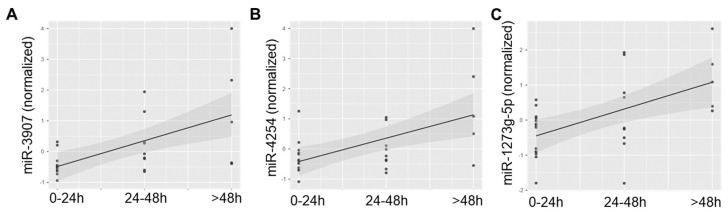
The scatter plots display quantile normalized levels of miR-3907 (**A**), miR-4254 (**B**), and miR-1273g-5p (**C**) in the saliva of children with sTBI across three timepoints: 0–24 h post-injury, 24–48 h post-injury, and >48 h post-injury. Mixed models controlling for within-subject effects showed that miR-3907 (*p* = 0.002), miR-4254 (*p* = 0.004), and miR-1273g-5p (*p* = 0.002) all increased over time. Trend lines with 95% confidence intervals are shown.

Next, we examined miRNA profiles in saliva samples from participants with sTBI taken < 24 h from the time of injury (*n* = 14), 24–48 h after injury (*n* = 9), and >48 h after injury (*n* = 5). Two-dimensional PLSDA achieved nearly complete separation of samples from each timepoint while accounting for 17.8% of the variance in the dataset (Figure 4A). The miRNAs with the greatest VIP scores tended to display low levels < 24 h after injury and increasing levels over subsequent time points (Figure 4B). Mixed models that used participant ID as a clustering variable (to control for within-subject effects) showed that the time since injury had a significant effect on salivary levels of miR-3907 (Est. = 0.640 (95% CI: 0.407–0.872; *p* = 0.002)), miR-4254 (Est. = 0.709 (95% CI: 0.282–1.136, *p* = 0.004)), and miR-1273g-5p (Est. = 0.766 (95% CI: 0.321–1.211, *p* = 0.002)) (Figure 5).

### 3.3. Pathway Analysis

**Figure 6:**
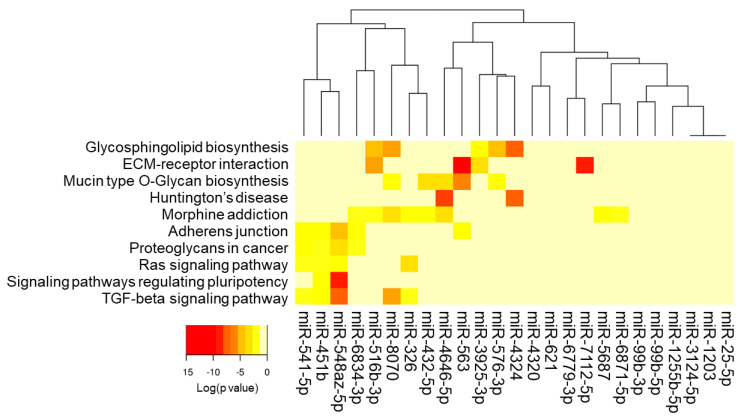
The heat map displays 10 pathway/miRNA clusters with significant (adj. *p* < 0.05) over-representation on pathways union analysis in DIANA miRPath. Among the 25 miRNA candidates, miR-541-5p, miR-451b, and miR-548az-5p all target ≥ four pathways with significant over-representation relative to that expected by chance alone.

Putative transcripts targeted by the 25 miRNA candidates that differentiated sTBI and control groups were explored with DIANA miRPath. These 25 miRNAs demonstrated target enrichment for 15 pathways on gene union analysis, including several brain-related targets: glioma (14 transcripts, seven miRNAs, *p* = 0.0075), amphetamine addiction (13 transcripts, nine miRNAs, *p* = 0.012), long-term depression (14 transcripts, eight miRNAs, *p* = 0.017), and glutamatergic synapse (19 transcripts, 12 miRNAs, *p* = 0.017) (Table 4). *SLC8A1* was targeted by four of the twenty-five miRNA candidates (*p* = 0.0037; miR-4320, miR-432-5p, miR-541-5p, miR-6871-5p). *GRIN2B* was targeted by seven of the 25 miRNA candidates (*p* = 0.0084; miR-3925-3p, miR-516b-3p, miR-541-5p, miR-548az-5p, miR-576-3p, miR-6779-3p, miR-6871-5p). Pathway union analysis identified 10 significant miRNA/pathway clusters (Figure 6).

## 4. Discussion

This study demonstrates that saliva miRNA profiles in children with sTBI may differ from peers with other trauma below the head and neck. In addition, several individual miRNAs appear to increase during the acute recovery phase after sTBI. Specifically, levels of miR-326 and miR-4646-5p were lower among children with sTBI, while levels of miR-1255b-5p, miR-3142, and miR-4320 were higher. Salivary levels of miR-3907, miR-4254, and miR-1273g all increased in the 48 h after sTBI. The putative gene targets of these candidate miRNAs involve a wide range of physiologic processes. Several brain-related pathways are over-represented (e.g., glutamatergic synapse, long-term depression), and specific molecular cascades implicated in TBI are also noted (e.g., TGF-beta signaling) [22].

Although several studies have examined miRNA levels in sTBI [8,9,13,23,24,25], to our knowledge, this is among the first to utilize saliva and to include pediatric patients. The importance of these distinctions is highlighted by the fact that the miRNA candidates identified in this study display minimal overlap with previous investigations. Identification of novel miRNAs in this study was also facilitated by the use of high-throughput sequencing technology, which allowed agnostic interrogation of over 2000 human miRNAs (as compared to targeted polymerase chain reactions used in prior studies of sTBI). Notably, there was also minimal overlap between the miRNA candidates in this study and the miRNAs identified in prior studies of mild TBI [25,26]. Physiologically, the injury response and recovery processes after sTBI are likely to differ significantly from mild TBI [27,28]. This appears to be reflected in miRNA profiles and the pathways they target.

Levels of miR-326 were reduced in children with sTBI relative to non-TBI controls. Previous studies have shown that miR-326 may be involved in the pathogenesis of hypoxic-ischemic brain damage in neonates [29]. Measurement of miR-326 as a diagnostic biomarker has previously been proposed for several brain-related conditions, including multiple sclerosis and glioma [30,31]. Similarly, miR-4646-5p, an sTBI biomarker candidate identified in this study, has previously been isolated in neuron-derived extracellular vesicles [32] and has been proposed as a potential biomarker for amyotrophic lateral sclerosis [33]. Finally, levels of miR-4320 have shown potential diagnostic utility for subarachnoid hemorrhage [34,35], while inhibition of miR-3142 has been implicated in dysregulated neuronal apoptosis associated with Alzheimer’s disease [36]. Together, these prior studies suggest that miRNA candidates identified in the present investigation have important roles in the pathophysiology of the central nervous system.

Understanding the biologic roles of each miRNA candidate is important for interpreting their expression patterns in the acute and sub-acute post-injury phases. For example, measuring miR-326, miR-4646-5p, miR-4320, miR-3142, and miR-1255b-5p in the immediate post-injury period could be a useful diagnostic tool for sTBI. However, specific elevations in miR-3142 or miR-326 may provide insights into more specific pathophysiology, such as subarachnoid hemorrhage or hypoxic ischemia of the central nervous system [29,34,35]. Such prognostic information about the severity and clinical trajectory of sTBI could aid clinical management. However, the findings from this initial exploratory study require validation in larger, independent cohorts prior to clinical application. To validate these findings, it will be important that future studies incorporate a wider variety of central nervous system injuries and employ long-term follow-up to assess sTBI-related morbidity.

In the current study, we explored the prognostic potential of salivary miRNA through hierarchical clustering analysis (Figure 2). The hierarchy dendrogram clearly separated control and sTBI participants. Participants 7, 9, and 10 had miRNA profiles that are quite closely related. Interestingly, all three suffered subdural hematomas. Likewise, participants 2 and 5 had very closely related miRNA profiles and were the only two participants to suffer subdural hematomas other than participants 7, 9, and 10 (albeit at a different trauma center). Finally, participants 11 and 12 revealed the two most closely related miRNA profiles. Not only did they both suffer subdural hematomas, but they each sustained injuries in motor vehicle collisions. Intriguingly, all of these participants displayed lower levels of miR-326.

The multi-site nature of this study is a strength; however, the subdural clustering described above illustrates how study site and collection technique could also impact miRNA patterns. Another limitation of this study is the lack of a healthy control group. Inclusion of healthy controls would have supported the premise that miRNA differences between the sTBI group and the group with injuries below the head/neck could be attributed to sTBI rather than injuries to other organ systems, such as the spleen or liver. Prior studies of serum miRNA biomarkers for pathology in the spleen (miR-18b-5p, miR-214, miR-452) [37,38], muscle (miR-206, miR-133a) [39], kidney (miR-21) [40], and liver (miR-122) [41] do not demonstrate overlap with the miRNAs identified in this study. However, future studies of sTBI biomarkers should consider the inclusion of a healthy control group and utilize a larger sample size to ensure sufficient statistical power and generalizability of the findings.

Although injury mechanism and symptom trajectory may have a large influence on saliva miRNA profiles, it is possible that other variables could also impact miRNA levels. There was a small age difference between the sTBI and control groups. As we have previously shown [42], age can impact salivary miRNA levels. Our analyses also did not control for the timing of intubation relative to sample collection, though longitudinal samples were routinely collected following oral hygiene procedures by nursing staff. Saliva miRNA levels can also be impacted by food intake and exercise. However, participants in this study were generally immobile and *nil per os* at the time of saliva collection. We acknowledge there was minimal diversity of functional outcomes and little morbidity in the sTBI cohort. This limited our ability to assess the relationship between long-term outcomes and saliva miRNA profiles and to examine the relationship of saliva miRNAs with the severity of TBI symptoms. Future studies should investigate these relationships in pediatric sTBI and leverage larger sample sizes to explore relationships between mechanisms of injury and miRNA patterns. Use of more rapid RNA quantification technology, such as quantitative polymerase chain reactions, will be necessary to measure a focused panel of candidate miRNAs and advance this technology toward clinical application.

## 5. Conclusions

To our knowledge, this is the first study to examine saliva miRNA perturbations in children with moderate-to-severe TBI. This is also the first study to describe longitudinal patterns in saliva miRNA in the acute post-injury period. The study’s statistical power raises the risk for false-positive results and limits conclusions about the veracity of specific miRNAs for detecting sTBI. Further studies, with larger sample sizes, are needed to confirm these findings and to interrogate whether these miRNA profiles could one day aid in prognostication following moderate-to-severe TBI.

## Figures and Tables

**Table 1:** Demographics of study participants.

	All (*n* = 23)	sTBI Group (*n* = 14)	Control Group (*n* = 9)
Sex (% male)	9 (39.1)	5 (35.7)	4 (44.4)
Age in months, mean (SD)	88.5 (63)	67.3 (52)	122.9 (64)
Weight in kg, mean (SD)	34.0	23.0 (14)	54.3 (39)

**Table 2:** Injuries sustained by sTBI patients.

Patient #	Trauma Center	Mechanism of Injury	Intracranial Injuries
1	A	fall	SAH, cerebral contusion
2		fall	SDH
3		fall	SAH
4		fall	EDH
5		MVC	SDH
6		car vs. pedestrian	none ^1^
7	B	fall	SDH
8		ATV ejection	EDH
9		MVC	SDH
10		fall	SDH
11		MVC	SDH
12		MVC	SDH, EDH
13	C	fall off bicycle	SAH
14		[data not reported]	[data not reported]

^1^ Patient qualified for study by Glasgow Coma Scale. Abbreviations: MVC = motor vehicle collision; ATV = all terrain vehicle; SAH = subarachnoid hemorrhage; SDH = subdural hemorrhage; EDH = epidural hemorrhage.

**Table 3:** Injuries sustained by control group patients.

Patient #	Trauma Center	Organs Injured
15	A	bone, spleen
16		bone, muscle, ligament, tendon
17		kidney
18		liver
19	B	[data not reported]
20		[data not reported]
21	C	spleen
22		bone
23		bone

**Table 4:** Kyoto Encyclopedia of Genes and Genomes (KEGG) pathways targeted by miRNAs.

Pathway	*p*-Value	Transcripts (#)	MiRNAs (#)
ECM receptor interaction	3.19 × 10^−12^	13	9
Proteoglycans in cancer	3.05 × 10^−5^	39	14
FoxO signaling pathway	0.00049	31	12
Insulin signaling pathway	0.0019	32	16
PI3K-Akt signaling pathway	0.0019	62	18
Glioma	0.0075	14	7
MAPK signaling pathway	0.0085	46	14
ErbB signaling pathway	0.0.011	19	10
Amphetamine addiction	0.012	13	9
N-Glycan biosynthesis	0.014	9	5
mTOR signaling pathway	0.014	16	10
Protein processing in ER	0.014	31	15
Long-term depression	0.017	14	8
Cell adhesion molecules	0.017	20	10
Glutamatergic synapse	0.017	19	12

## Data Availability

The raw RNA sequencing results are available as a supplemental file.

## Supplementary Materials

The following supporting information can be downloaded at: https://www.mdpi.com/article/10.3390/jcm13175065/s1, Table S1: Study data.
